# Volumetric registration framework for multimodal functional magnetic resonance and optoacoustic tomography of the rodent brain

**DOI:** 10.1016/j.pacs.2023.100522

**Published:** 2023-06-09

**Authors:** Irmak Gezginer, Zhenyue Chen, Hikari A.I. Yoshihara, Xosé Luís Deán-Ben, Daniel Razansky

**Affiliations:** a Institute for Biomedical Engineering and Institute of Pharmacology and Toxicology, Faculty of Medicine, University of Zurich, Switzerland; b Institute for Biomedical Engineering, Department of Information Technology and Electrical Engineering, ETH Zurich, Switzerland

**Keywords:** Optoacoustic tomography, Magnetic resonance imaging, Multimodal imaging, Image registration, Mouse brain template, Mouse brain atlas

## Abstract

Optoacoustic tomography (OAT) provides a non-invasive means to characterize cerebral hemodynamics across an entire murine brain while attaining multi-parametric readouts not available with other modalities. This unique capability can massively impact our understanding of brain function. However, OAT largely lacks the soft tissue contrast required for unambiguous identification of brain regions. Hence, its accurate registration to a reference brain atlas is paramount for attaining meaningful functional readings. Herein, we capitalized on the simultaneously acquired bi-modal data from the recently-developed hybrid magnetic resonance optoacoustic tomography (MROT) scanner in order to devise an image coregistration paradigm that facilitates brain parcellation and anatomical referencing. We evaluated the performance of the proposed methodology by coregistering OAT data acquired with a standalone system using different registration methods. The enhanced performance is further demonstrated for functional OAT data analysis and characterization of stimulus-evoked brain responses. The suggested approach enables better consolidation of the research findings thus facilitating wider acceptance of OAT as a powerful neuroimaging tool to study brain functions and diseases.

## 1 Introduction

Labelling and parcellation of whole-brain images is essential to advance our understanding of brain functions. Registration of the reconstructed images to a reference anatomical brain atlas is commonly performed in functional magnetic resonance imaging (fMRI) for investigating the structure-function relationship of brain circuits [1]. Brain atlases have facilitated the interpretation of fMRI data and the integration of information contained in the images in standard spatial coordinates [2]. This in turn serves as a basis for the development of new neuroimaging approaches operating at the whole-brain level. Recently, optoacoustic tomography (OAT) has been advanced as a non-invasive approach to map multiple hemodynamic parameters across the entire murine brain [3], [4], [5]. The versatile contrast and high spatio-temporal resolution [6] of OAT provide a powerful means to retrieve structural, functional, molecular and kinetic information growingly exploited in both clinical [7], [8] and preclinical [9], [10] settings. OAT can visualize endogenous contrast, e.g. from hemoglobin [11], melanin [12], or lipids [13], as well as exogenous contrast agents [14], [15], which makes it an attractive tool for a large variety of small animal and clinical applications [9], [16], [17], [18], [19], [20], [21]. Of particular importance is the distinct optical absorption spectrum of oxygenated (HbO) and deoxygenated hemoglobin (HbR). These two molecules abundantly present in mammalian tissues facilitate the visualization of cerebral vasculature, and, more importantly, enable the real-time brain-wide multi-parametric readouts of cerebral hemodynamics in vivo not readily available with fMRI or other modalities [4], [22], [23], [24]. The strong contrast from blood overshadows the weaker contrast generated by other brain tissues, which hinders anatomical mapping of brain subregions and clear delineation of their boundaries. A robust methodology to efficiently register the reconstructed images to a reference brain atlas is then essential to fully exploit the unique capabilities of OAT for functional neuroimaging.

The feasibility of simultaneous measurements of brain hemodynamics with a hybrid magnetic resonance optoacoustic tomography (MROT) system has recently been demonstrated [3], [5]. OAT offers versatile functional contrast at high volumetric imaging rate [25], [26], [27], [28], whereas the excellent soft tissue contrast of MRI complements the lack of anatomical information in OAT images. Accurate registration between MRI and OAT images is essential to integrate the information extracted from both modalities. Registration of images acquired independently from the standalone modalities has previously been done with landmark-based approaches [29], [30], automated registration algorithms [31], and deep-learning-based methods [32]. Cross-sectional (2D) OAT images have been considered for this purpose, which could not be reliably matched to the corresponding MRI slices due to lack of common landmarks. Even when employing volumetric (3D) OAT data, vascular anatomy differs significantly between mice, while differences in orientation and tissue deformations resulting from independent acquisitions further hamper image transformation into the standard spatial coordinates (common space) defined by a reference brain atlas. The recent development of a truly hybrid MROT system significantly reduced the problem’s complexity to rigid-body transformations whereas the vascular networks resolved in the OAT image could be matched to a magnetic resonance angiography (MRA) image by concurrent bi-modal image acquisition without employing additional deformations [3], [5]. However, the high degree of multi-modal system integration has been achieved with MROT at the expense of sub-optimal OAT imaging performance, providing an advantage for standalone systems not geometrically constrained to fit inside the MRI scanner bore. Also, positioning the animal and maintaining it in a physiologically-relevant condition is more straightforward with standalone measurements.

In this work, we systematically approach the anatomical registration of images acquired by a standalone OAT modality to the anatomical brain atlas by capitalizing on the information provided by the MROT scanner. A custom OAT template of the mouse brain has been constructed in the common space considering a set of bi-modal MROT images. The template is subsequently used as a reference to spatially normalize (i.e., transform into a common space) OAT images acquired with a different (standalone) system. The enhanced functional OAT imaging performance is demonstrated experimentally for brain responses to peripheral stimulation in mice.

## 2 Materials and methods

### 2.1 Hybrid magnetic resonance optoacoustic tomography (MROT) system

**Fig. 1:**
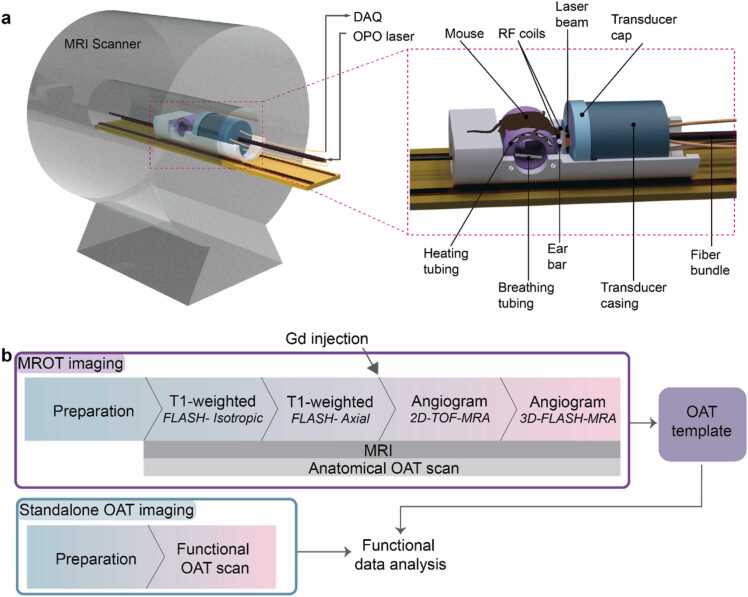
Hybrid magnetic resonance optoacoustic tomography (MROT) imaging. a Layout of the scanner enabling simultaneous MRI and OAT readings from the murine brain. The main components of the OAT module are shown in the inset. b Schematic representation of the bi-modal and standalone in vivo imaging protocols. The animal and all the hardware modules remain stationary throughout the MROT imaging. In the case of standalone OAT imaging, an OAT template generated using the MROT imaging data is employed to facilitate functional data analysis.

Hybrid MROT imaging was realized by inserting a volumetric OAT system into a 9.4 T MRI scanner (BioSpec 94/20, Bruker BioSpin, Germany), as described elsewhere [3], [5]. Briefly, the OAT module consists of an MRI-compatible spherical matrix transducer array (Imasonic SAS, Voray, France), an MRI-compatible fiber bundle (CeramOptec GmbH, Bonn, Germany) and customized RF coils (Fig. 1**a**). A short-pulsed (<10 ns) optical parametric oscillator (OPO) laser (Spit-Light, Innolas Laser GmbH, Germany) was used for the optoacoustic signal excitation. The light pulses carrying ∼8 mJ of energy were delivered to the tissue surface via a fiber bundle protruded through a central 8 mm aperture of the array. The array consists of 384 piezocomposite elements with central frequency 5 MHz (>80% bandwidth) distributed on a hemisphere with a radius of 40 mm and angular aperture of 130 degrees. The spatial resolution of the array was characterized as 163.5 µm and 163.2 µm along the lateral (x,y) and axial (z) dimensions, respectively [3], [5]. To provide acoustic coupling, the volume between the spherical surface of the array and tissue surface was filled with deuterium oxide (heavy water) and sealed with a custom non-magnetic polyetheretherketone (PEEK) cap. An optically and acoustically transparent thin polyethylene membrane was glued to a 36 mm central opening in the cap. Coupling between the mouse skin and the membrane was facilitated with acoustic gel enriched with heavy water. A pair of customized RF coils, with polarization perpendicular to the main magnetic field, was integrated on both sides of a 3D-printed cylindrical animal holder. The holder allows placing the animal in a supine position with its nose pointing downwards and the brain located within the central region of the spherical array geometry. The generated optoacoustic signals were sampled with a custom-made data acquisition system (DAQ, Falkenstein Mikrosysteme GmbH, Germany) at 40 mega samples per second (Msps).

### 2.2 Hybrid MROT imaging

In total, *N* = 13 mice were imaged with the MROT system. For anatomical imaging (*N* = 11), anesthesia was induced in an induction box (4% isoflurane in a 20%/80% O_2_/air mixture) and maintained via a breathing mask (1.5% isoflurane in a 20%/80% O_2_/air mixture) in the animal holder. Both the mouse scalp and skull were kept intact for the experiments. A mouth clamp and a 3D-printed stereotactic frame ensured immobilization of the mouse head. The OAT module was inserted into the MRI bore after positioning the mouse. Physiological status of the mouse was continuously monitored with a rectal thermometer and an MRI-compatible pulse oximeter (PhysioSuite, Kent Scientific Corporation, USA) connected to the paw. Body temperature was stabilized to ∼37 °C using a tunable water heating unit. The bi-modal data acquisition was performed concurrently. For OAT, the laser wavelength was rapidly switched between five different values (700 nm, 730 nm, 755 nm, 800 nm, and 850 nm) at a pulse repetition frequency of 50 Hz. In parallel, isotropic whole-brain T1-weighted (3D FLASH, TR/TE = 150/5 ms, flip angle = 20°, 200 µm isotropic resolution, FOV= 20 × 20 × 8 mm^3^, 4 averages) and axial T1-weighted (FLASH, TR/TE = 500/5 ms, flip angle = 30°, resolution= 125 × 250 µm^2^, FOV= 20 × 20 mm^2^, slice thickness= 0.3 mm, 20 slices, 10 averages) MRI images were acquired. Contrast-enhanced 2D time-of-flight (TOF) MR angiography (2D-TOF-MRA, TR/TE = 13/1.89 ms, flip angle = 80°, resolution= 250 × 250 µm^2^, FOV= 20 × 20 mm^2^, slice thickness= 0.3 mm, 20 slices, 16 averages) covering the same volume as the axial T1-weighted MRI, and contrast-enhanced 3D FLASH MR angiography (3D-FLASH-MRA, TR/TE = 18/2 ms, flip angle = 20.07°, 125 µm isotropic resolution, FOV= 20 × 16 ×12.8 mm^3^, 8 averages) images were acquired after administration of gadolinium (Gd) based contrast agent (Dotarem, Guerbet, 0.5 mmol/mL, 40 μl) (Fig. 1b).

Raw OAT signals were bandpass filtered between 0.1 and 8 MHz with the images for each excitation wavelength reconstructed using a filtered back-projection algorithm [33] (100 ×100 ×100 µm^3^ voxel size, 8 ×8 ×4 mm^3^ FOV). During the reconstruction, different speed of sound values were considered for the heavy water medium versus the mouse tissue in order to enhance the OAT image quality [3], [5]. HbO and HbR distributions were estimated using linear spectral unmixing [4]. The total hemoglobin (HbT) was calculated as the sum of HbO and HbR whereas oxygen saturation (sO_2_) was defined as the HbO to HbT ratio.

For functional imaging (*N* = 2), mice were anesthetized with intraperitoneal (IP) injection of a combination of ketamine (100 mg/kg body weight, Pfizer) and xylazine (10 mg/kg bodyweight, Bayer). Two injections with a 5 min gap were performed for induction to prevent cardiac depression. Maintenance injection with a combination of ketamine (25 mg/kg body weight) and xylazine (1.25 mg/kg body weight) was administered every 45 min. Mice were positioned and monitored in the same manner as for anatomical MROT imaging. Functional MRI and OAT data were acquired during an electrical stimulation paradigm (frequency = 4 Hz, pulse width = 0.5 ms, intensity= 0.5 mA, 80 s baseline, 20 s stimulation, 80 s interstimulus interval, 9 stimulation cycles, 980 s duration) applied to the left hindpaw of the mouse. Stimuli were sent using a constant current isolator (Model A365R, World Precision Instruments, USA). Synchronization of the electrical stimulation with the light pulses and the concurrent MROT acquisition was achieved with an external trigger device (Pulse Pal V2, Sanworks, USA). Blood oxygenation level-dependent (BOLD) data were acquired using gradient-echo echo-planar imaging sequence (GE-EPI, TR/TE = 995/12 ms, flip angle = 60°, resolution= 250 × 250 µm^2^, FOV= 20 × 10 mm^2^, slice thickness= 0.7 mm, 11 slices, 1 average) with an effective temporal resolution of 1 s for volumetric acquisition. Volumetric time-lapse OAT data were recorded in the same manner as for the anatomical MROT imaging. Raw signal matrices were downsampled to 1 Hz via signal averaging and OAT images were reconstructed following the same procedure as for anatomical MROT imaging.

### 2.3 Standalone OAT measurements

Performance of the proposed methodology was validated with a standalone OAT scanner optimized for functional whole-brain imaging in mice. It employs a spherical matrix transducer array (Imasonic SaS, Voray, France) consisting of 512 piezocomposite elements with a central frequency of 7 MHz (>80% bandwidth) spread over a hemispherical surface covering an angular aperture of 150 degrees. The spatial resolution of the array was characterized as 113 µm and 113 µm along the lateral (x,y) and axial (z) dimensions, respectively [4]. The nanosecond laser pulses were delivered to the mouse head from four different directions to improve on uniformity of the light delivery into the brain. The generated optoacoustic signals were digitized with the same data acquisition system that was used for the hybrid MROT system.

In total, *N* = 17 mice were imaged with the standalone OAT system. Mice were anesthetized with IP injection of a combination of ketamine (100 mg/kg body weight, Pfizer) and xylazine (10 mg/kg bodyweight, Bayer). Two injections with a 5 min gap were performed for induction to prevent cardiac depression. Maintenance injection with a combination of ketamine (25 mg/kg body weight) and xylazine (1.25 mg/kg body weight) was administered every 45 min. The mouse was placed on a 3D translation stage in supine position with the nose pointing forward and immobilized using a custom stereotactic frame and a mouth clamp. A 20%/80% O_2_/air mixture was supplied via a breathing mask during the experiment. Physiological status of the mouse was monitored and stabilized in the same manner as for the hybrid MROT imaging. Volumetric OAT images of each mouse were acquired by switching the laser wavelength between five different values (700 nm, 730 nm, 755 nm, 800 nm, and 850 nm) on a per-pulse basis at a pulse repetition frequency of 100 Hz. Raw signal matrices were downsampled to 1 Hz via signal averaging and OAT images were reconstructed following the same procedure as for MROT imaging. For functional OAT (*N* = 1), volumetric time-lapse OAT data were acquired during an electrical stimulation paradigm (Fig. 1b, frequency = 4 Hz, pulse width = 5 ms, intensity= 0.5 mA, 82 s baseline, 8 s stimulation, 82 s interstimulus interval, 4 stimulation cycles, 440 s duration) applied to the left hindpaw of the mouse. Stimuli were sent using a constant current isolator (Model A365R, World Precision Instruments, USA). Synchronization of the light pulses and the electrical stimulation was achieved with an external trigger device (Pulse Pal V2, Sanworks, USA).

### 2.4 Animal models

Athymic female nude mice (Foxn1^nu^, Charles River Laboratories, Germany, 9–11 week-old, *N* = 13) were imaged with the hybrid MROT system. Female GCaMP6f mice (C57BL/6 J-Tg(Thy1-GCaMP6f) GP5.17Dkim/J, the Jackson Laboratory, USA, 6–11 week-old, *N* = 8) and athymic nude mice (6–11 week-old, *N* = 9) were imaged with the standalone OAT system. All animals were housed in individually ventilated, temperature-controlled cages under a 12-hour reversed dark/light cycle. Pelleted food (3437PXL15, CARGILL) and water were provided ad-libitum. All animal procedures were conducted in accordance with the Swiss Federal Act on Animal Protection and were approved by the Cantonal Veterinary Office Zurich.

### 2.5 OAT template of the mouse brain

**Fig. 2:**
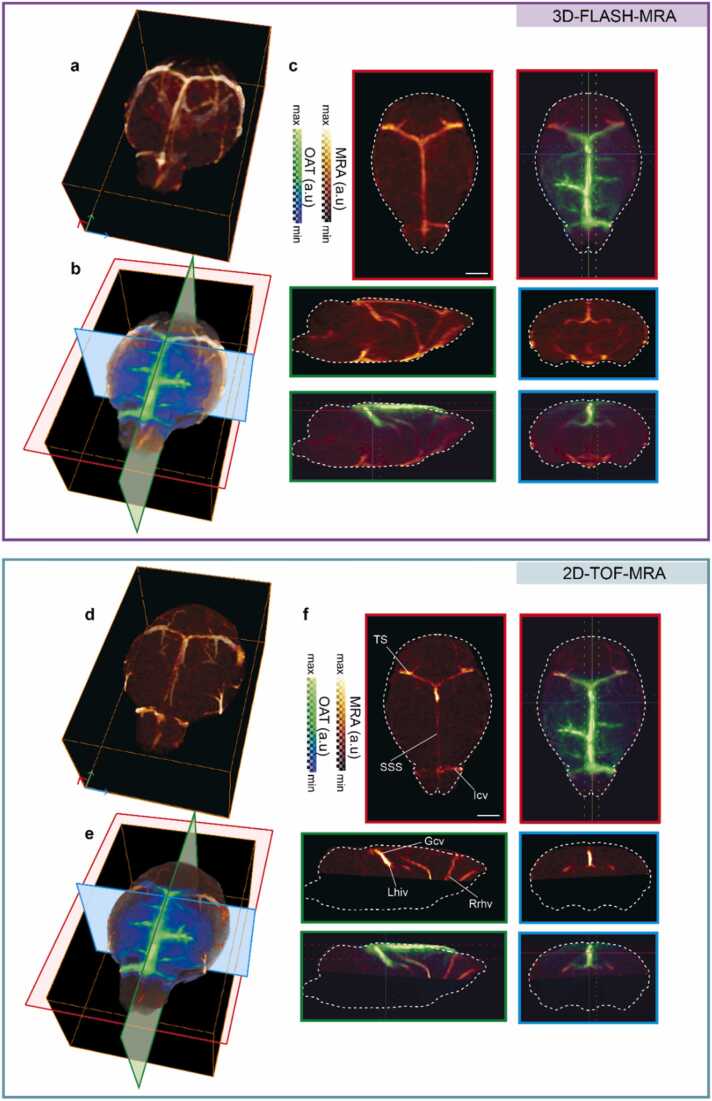
Coregistration of OAT and contrast-enhanced MRA images acquired with the hybrid MROT scanner. a Volumetric 3D-FLASH-MRA image of the mouse brain. b Overlay of the OAT volume onto the 3D-FLASH-MRA image after coregistration. c Axial, sagittal and coronal views of the 3D-FLASH-MRA image superposed onto the coregistered OAT image. Images are shown as maximal intensity projections (MIPs) of 0.8 mm thick volumes centered around the slices shown in b. d Volumetric 2D-TOF-MRA image of the mouse brain. e Overlay of the OAT volume onto 2D-TOF-MRA image after coregistration. f Axial, sagittal and coronal views of 2D-TOF-MRA image superposed to the coregistered OAT image. Images are shown as MIPs of 0.8 mm thick volumes centered around the slices shown in **e**. All scale bars: 2 mm.

**Fig. 3:**
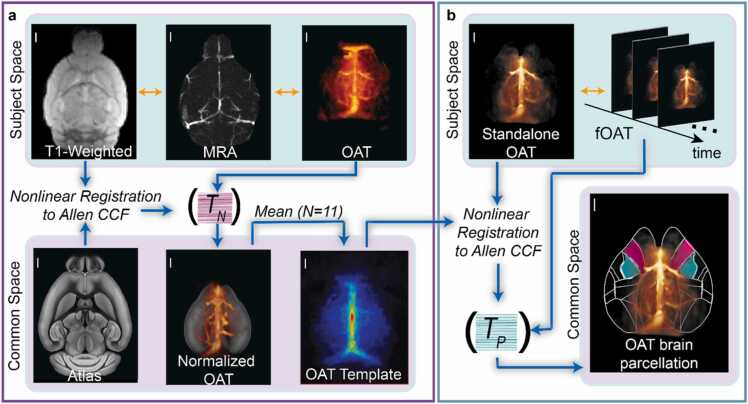
Flowchart displaying the main data processing steps involved in creating the OAT template. Orange arrows indicate coregistration between images. All registration processes were performed automatically after an initial manual alignment. a Construction of the custom mouse brain template for OAT. First, subsequent alignments of the T1-weighted MRI image with the MRA, and MRA with the OAT were achieved, resulting in coregistration between the T1-weighted MRI and OAT images. The coregistration process was performed in subject space of an individual animal. Next, the T1-weighted image of each mouse was skull-stripped and spatially normalized to the Allen CCF. The nonlinear spatial transformation matrix computed for individual mice (T_N_) was applied to the OAT to transform it onto the CCF, followed by intensity normalization and averaging across mice (N = 11), finally resulting in the custom OAT template of the mouse brain. b Brain parcellation of standalone OAT data. Coregistration between an OAT image acquired by an arbitrary standalone system and the template was performed via affine transformations. The transformation matrix computed during this coregistration (T_P_), was used to normalize the remaining frames of the OAT data to the common space defined by Allen CCF. Brain parcellation of the functional OAT data was done considering anatomical boundaries defined by the Allen brain atlas. All scale bars: 1 mm.

**Fig. 4:**
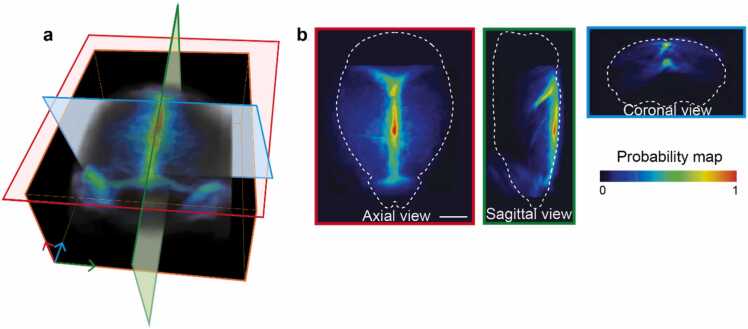
OAT mouse brain template. a Volume rendered image of the template. b Axial, sagittal and coronal views of the template. Images are shown as MIPs of a 0.8 mm thick volume centered around the slices shown in a. All scale bars: 2 mm.

Images acquired with the hybrid system were used for constructing an OAT template of the mouse brain. Specifically, MRA, T1-weighted (T1w), and OAT images were considered for each mouse (See Section 2.2 for details). First, coregistration between T1w and MRA images (2D-TOF-MRA or 3D-FLASH-MRA) was realized using rigid transformations after an initial manual alignment (pre-registration) (Fig. 2). Note that a separate axial T1-weighted image (T1w-axial) covering the same space as 2D-TOF-MRA was additionally employed for registering T1w with 2D-TOF-MRA due to the lack of soft tissue contrast of 2D-TOF-MRA. Next, MRA images were coregistered with OAT images using mutual information as the similarity measure, making use of the vascular contrast in both images. This two-step process resulted in coregistration between T1w and OAT images (SFig. **1**). T1w images were subsequently skull stripped and normalized to the Allen Institute of Brain Science (AIBS) common coordinate framework (CCF) [34], resulting in a spatial transformation matrix (*T*_*N*_) which was further used to normalize the OAT image to the Allen CCF for each mouse. Normalized OAT images were smoothed with a Gaussian kernel with full-width-at-half-maximum (FWHM) of 0.1 mm, intensity normalized, and averaged for all mice (*N* = 11) to construct the OAT template (Figs. 3a, 4). Next, a standalone OAT image (or a single-frame image from time-lapse functional OAT data) was manually aligned with the in-house template using rigid transformations. Subsequently, the OAT image was normalized to the template via affine transformations with Amira (Thermo Fisher Scientific Inc., Waltham, MA, USA), resulting in a spatial transformation matrix (T_P_). Since the OAT template is inherently coregistered with the Allen brain atlas, the remaining frames of the functional data were normalized to the common space using the obtained spatial transformation matrix (Fig. 3b).

### 2.6 Registration of standalone OAT data to the template

**Fig. 5:**
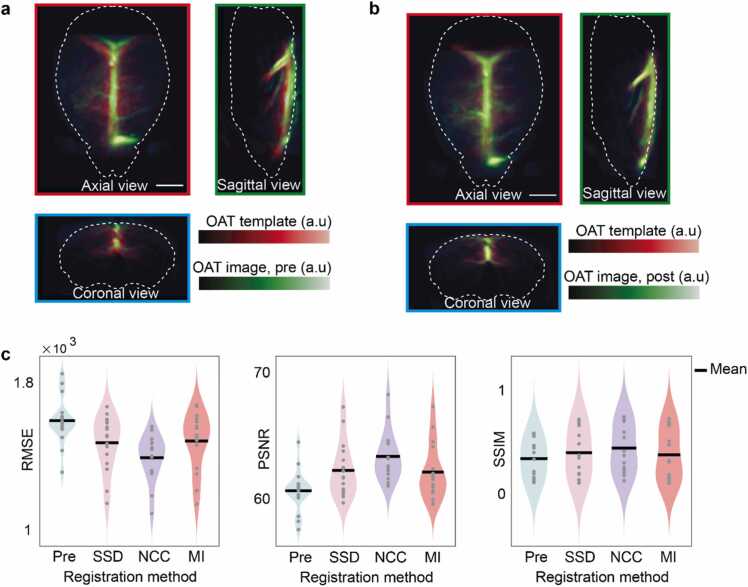
Coregistration to the OAT template. a Representative overlay of an arbitrary OAT image and the template after manual coregistration. b Corresponding overlay after subsequent automatic coregistration using affine transformations with NCC as the similarity index. c Evaluation of registration performance for SSD, NCC and MI registration methods. RMSE, PSNR and SSIM performance metrics were computed after manual pre-alignment and automatic registration of the OAT images (*N* = 16). All scale bars: 2 mm.

**Fig. 6:**
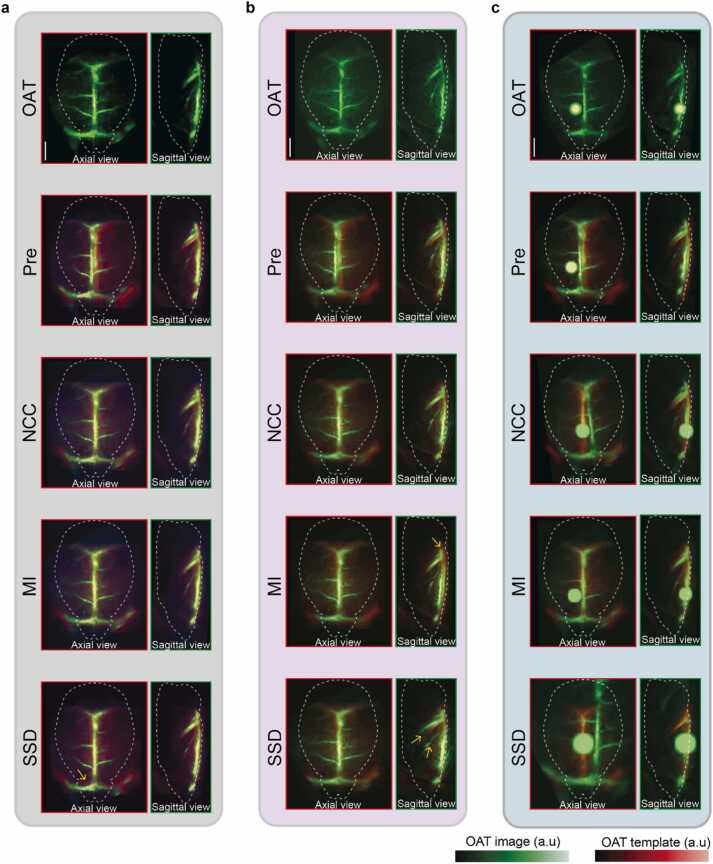
Registration of OAT images with various characteristics to the OAT template via NCC, MI and SSD methods. OAT images reconstructed with model-based methods (a), reconstructed with backprojection (b), and exhibiting a high-intensity artifact (c) were considered. Same initial manual alignment was applied to all OAT images (*P* *re* ). Yellow arrows indicate locations of registration mismatch. All scale bars: 2 mm.

Registration of the standalone OAT images to the template was defined as an optimization problem where a similarity measure (cost function) was maximized (or minimized) by aligning the images to result in the best spatial correspondence. This was realized using custom Matlab (MathWorks Inc., Natick, MA, USA) scripts, SPM12 (Wellcome Trust Centre for Human Neuroimaging, University College London) and Amira. First, the OAT image of each mouse was manually aligned with the template using the SPM12-based image to serve as an initial solution to the optimization problem and facilitate convergence of the algorithm [35] (Fig. 5a and 6). The pre-aligned OAT image was subsequently aligned with the template using 9-degree of freedom (9-dof) affine transformations (i.e., 3 translations + 3 rotations + 3 anisotropic scaling parameters) with Amira (Fig. 5b). Intensity-based similarity measures were examined for the nonlinear coregistration (normalization) process. Specifically, we used sum of squared distances (SSD), normalized cross-correlation (NCC) and mutual information (MI) as possible similarity measures (registration methods). SSD assumes the voxel intensity from corresponding images should have identical intensity values, which is calculated as

$$\mathrm{SSD}=\sum_{x=1}^{R_{x}}\sum_{y=1}^{R_{y}}\sum_{z=1}^{R_{z}}{\left|A\left(x,y,z\right)-B(x,y,z)\right|}^{2},$$

where A(x,y,z) and B(x,y,z) are corresponding voxel intensities of the 3D images and $R_{x}$*,* $R_{y}$*,* and $R_{z}$ are the voxel numbers along each dimension. The SSD is being minimized during the optimization process. NCC registration is based on a linear relationship between intensities of the images, calculated via

$$\mathrm{NCC}=\frac{\sum_{x=1}^{R_{x}}\sum_{y=1}^{R_{y}}\sum_{z=1}^{R_{z}}\left[A\left(x,y,z\right)-\overset{®}{A}(x,y,z)\right]\left[B\left(x,y,z\right)-\overset{®}{B}(x,y,z)\right]}{\sqrt{\sum_{x=1}^{R_{x}}\sum_{y=1}^{R_{y}}\sum_{z=1}^{R_{z}}{\left[A\left(x,y,z\right)-\overset{®}{A}(x,y,z)\right]}^{2}}\sqrt{\sum_{x=1}^{R_{x}}\sum_{y=1}^{R_{y}}\sum_{z=1}^{R_{z}}{\left[B\left(x,y,z\right)-\overset{®}{B}(x,y,z)\right]}^{2}}},$$

where $\overset{®}{A}(x,y,z)$ and $\overset{®}{B}(x,y,z)$ are the mean intensity values of the images. The value of the NCC, ranging from 0 to 1, was maximized during the optimization process. MI registration is based on the Shannon entropy expressed via

$$\mathrm{MI}=H\left(A\right)+H\left(B\right)-H(A,B),$$

where $H\left(A\right)$ and $H\left(B\right)$ represent entropy of the corresponding image and H(A,B) is the joint entropy associated to the alignment of images. MI was maximized during the coregistration. While SSD and NCC are the most suitable measures for intramodal image registration, MI can be applied both for intramodal and intermodal registration [36]. After successful normalization, the standalone OAT image lies in the common space (Allen CCF) and thus can be parcellated with respect to the well-established anatomical brain atlas. Evaluation metrics, such as root mean square error (RMSE), peak signal to noise ratio (PSNR) and structural similarity index metric (SSIM) [37], were calculated before and after the automatic coregistration to quantitatively assess the normalization performance (Fig. 5**c**). RMSE uses the intensity differences between images, computed via

$$\mathrm{RMSE}=\sqrt{\frac{\mathrm{SSD}}{R_{x}R_{y}R_{z}}}\;$$

for images with voxel resolution of $R_{x}$ *x* $R_{y}$ *x* $R_{z}$. Smaller values of RMSE indicate a better registration as it reflects the error between images. PSNR, which is the more commonly used evaluation metric, expresses the ratio between maximum value of an image and the background noise. It was calculated as

$$\mathrm{PSNR}=10\log{\left(\frac{\max\left(B\right)}{\mathrm{RMSE}}\right)}^{2}\;,$$

where B is the reference image. Larger PSNR values indicate better registration performance. Finally, SSIM is a metric quantifying registration quality by measuring the perceptual difference between images and includes luminance masking and contrast masking terms. It assumes spatial dependencies and was calculated as

$$\mathrm{SSIM}=\frac{\left(2\mu_{a}\mu_{b}+C_{1}\right)\left(2\sigma_{\mathrm{ab}}+C_{2}\right)}{\left(\mu_{a}^{2}+\mu_{b}^{2}+C_{1}\right)\left(\sigma_{a}^{2}+\sigma_{b}^{2}+C_{1}\right)},$$

where $\mu_{a}$ is the mean of image A, $\mu_{b}$ is the mean of image B, $\sigma_{a}^{2}$ is the variance of image A, $\sigma_{b}^{2}$ is the variance of image B, $\sigma_{\mathrm{ab}}$ is the covariance of the images. $C_{1}$ and $C_{2}$ are small positive constants to avoid instability, which are dependent on the dynamic range of the images.

### 2.7 Functional data analysis

**Fig. 7:**
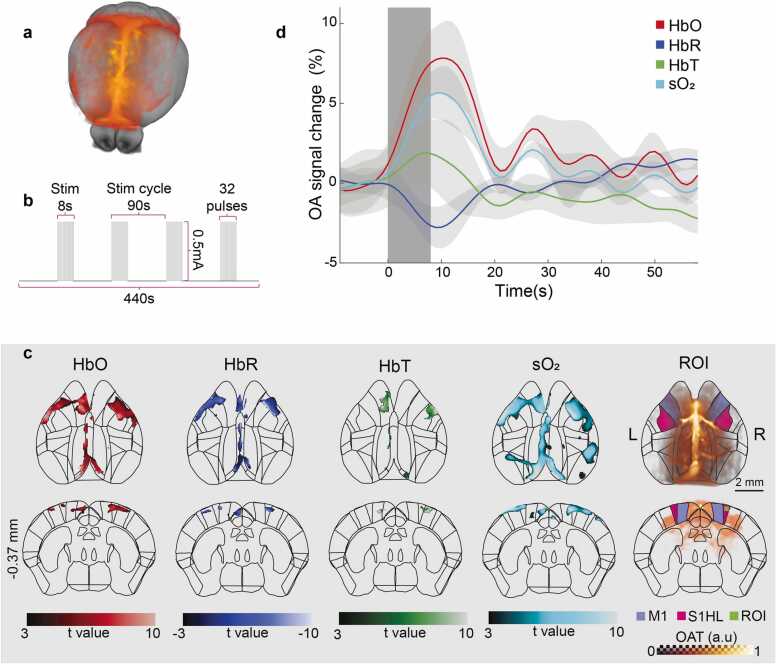
Sensory responses and brain partitioning for functional OAT data from a representative mouse. a Volume rendered overlay of OAT data on the Allen mouse brain atlas following normalization to CCF using the template. b Electrical stimulation paradigm applied to the left hindpaw of the mouse. c Stimulus-evoked responses of the HbO, HbR, HbT, and sO_2_ hemodynamic components. Activation maps (p_FWE_<0.05) were overlaid onto the mouse brain atlas. The rightmost column incorporates ROI definitions and overlay of the HbO component image on corresponding axial and coronal brain slices. M1, primary motor cortex; S1HL, primary somatosensory cortex hindlimb area. Coordinates of the coronal slice refer to the bregma distance in mm. d Averaged percent signal change of each hemodynamic component following sensory stimulation. Activation curves were computed from a 0.3 × 0.3 × 0.3 mm^3^ ROI in the contralateral S1HL, as indicated in panel c. The gray shaded area indicates the stimulation period.

Functional OAT and MRI data were preprocessed and analyzed using custom Matlab scripts, SPM12 and Amira. All reconstructed OAT images were spectrally unmixed (resulting in HbO, HbR, HbT and sO_2_ components), converted to NIFTI format, motion corrected, and smoothed by a Gaussian kernel with FWHM of 0.3 mm (3 × voxel size). All hemodynamic channels of the functional OAT data were normalized to the common space by applying the transformation matrix computed from a single-frame OAT image of the unmixed HbO component (Fig. 7**a,** see Section 2.6 for details). The fMRI data were corrected for motion, smoothed by a Gaussian kernel with FWHM of 0.6 mm, and spatially normalized to the Allen CCF.

**Fig. 8:**
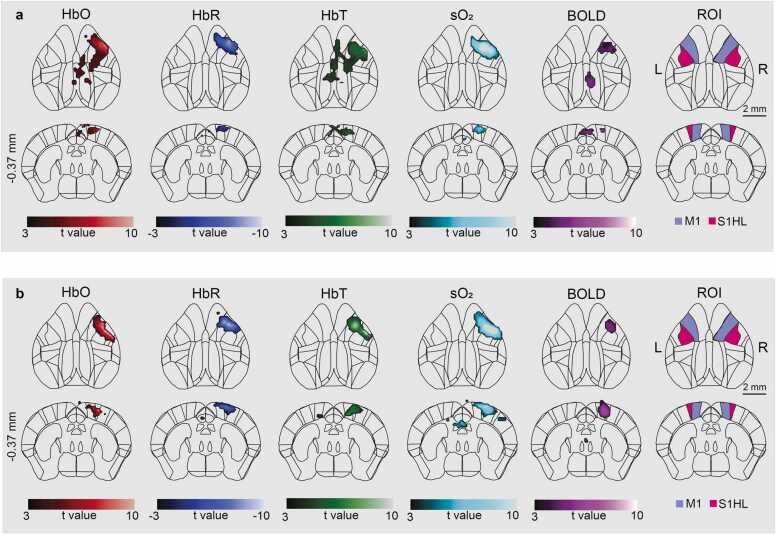
Concurrent functional OAT and MRI imaging with the MROT system. Stimulus-evoked responses of the HbO, HbR, HbT, sO_2_, and BOLD hemodynamic components calculated from Subject01 (a) and Subject02 (b). Activation maps (p_FWE_<0.05) were overlaid onto the mouse brain atlas. The rightmost column incorporates ROI definitions. M1, primary motor cortex; S1HL, primary somatosensory cortex hindlimb area. Coordinates of the coronal slice refer to the bregma distance in mm.

Normalized functional image data of each unmixed signal component (i.e., HbO, HbR, HbT, sO_2_, BOLD) was analyzed using a general linear model (GLM). The stimulation paradigm was first convolved with the hemodynamic response function (HRF) and used as a regressor for the first-level analysis. Default HRF parameters in SPM12 were modified with parameters optimized with characteristic small animal BOLD response [38]. Motion parameters obtained from the preprocessing step were used as nuisance regressors. Statistically significant activation maps (t-value maps) of the different hemodynamic components were obtained using a family-wise error (FWE) correction at p_FWE_< 0.05 after an initial threshold of p < 0.001 uncorrected. Since the normalized functional OAT images were in the Allen CCF, the t-value maps were simply overlaid with regions of interest (ROI) drawn based on the Allen mouse brain atlas (Fig. 7**c,** Fig. 8). Stimulus-evoked responses in the activated somatosensory area were extracted from the activated somatosensory area and the activation time courses were obtained by averaging the stimulation cycles, calculated by normalizing to the 10 s pre-stimulation baseline, and plotted as mean ± SEM (Fig. 7d).

## 3 Results

*In vivo* OAT and T1-weighted MRI imaging was performed in parallel after positioning the mouse in the MROT system (Fig. 1a, see Section 2.2 for details). The MRA images were subsequently acquired following tail-vein administration of Gd-based contrast agent (Fig. 1b). OAT-MRI coregistration was performed using a semi-automatic protocol (SFig. 1). First, the whole-brain anatomical MRI image (T1w) of each animal (*N* = 11) was aligned with the corresponding MRA image. The contrast of 2D-TOF-MRA images mainly stems from the blood vessels with the 3D-FLASH-MRA images providing additional soft-tissue contrast while covering the whole brain (Fig. 2). To suppress high-intensity signals from deeper brain vessels, 2D-TOF-MRA images were acquired from a relatively small brain volume covering superficial regions. T1w and 3D-FLASH-MRA images were skull stripped and aligned using rigid transformations and MI similarity metric. Due to the lack of soft tissue contrast in 2D-TOF-MRA images, an anatomical scan in the axial plane (T1w-axial) was additionally acquired. Note that T1w-axial lies in the same coordinate plane as the 2D-TOF-MRA, thus both images are automatically aligned. As a result, the transformation matrix computed by aligning T1w and T1w-axial images was applied for aligning between the 2D-TOF-MRA and T1w images. It was also possible to register between the T1w and 2D-TOF-MRA images acquired without administration of the contrast agent, yet by chiefly relying on manual alignment owing to suboptimal signal-to-noise ratio (SNR) (SFig. 2).

Due to the strong optical absorption contrast of hemoglobin as compared to surrounding tissues, the OAT images mainly depict the brain vessels (Fig. 2). The latter serve as landmarks for coregistration between OAT and MRA images, which could be done using rigid transformations without employing additional deformations by capitalizing on the multi-modal MROT imaging. The coregistration process ultimately results in aligned OAT, MRA and T1w images. Spatial alignment of vascular structures is readily recognized in the axial, sagittal and coronal views after overlaying the coregistered OAT image onto the 3D-FLASH-MRA image (Fig. 2c). A similar overlap of major vessels for 2D-TOF-MRA was observed for superior sagittal sinus (SSS), transverse sinus (TS), inferior cerebral vein (Icv), rostral rhinal vein (Rrhv), vein of Galen (Gcv), and longitudinal hippocampal vein (Lhiv) (Fig. 2 f). All coregistration steps were performed in the subject space defined by the coordinates of the aligned OAT-MRI images acquired with the hybrid MROT system, which were subsequently employed to build a custom OAT image template coregistered with the Allen brain atlas (Fig. 3a). This was achieved by spatial normalization of the bi-modal images taken from individual mice (*N* = 11) to the common space defined by the Allen CCF. Specifically, T1w images of individual mice were normalized to the CCF using affine transformation matrices (*T*_*N*_) computed separately for each animal. OAT were spatially normalized from the subject space to the CCF using *T*_*N*_ and then averaged to generate the brain template (Figs. 3a, 4). The OAT template was further normalized to its maximum intensity to render a probability map of vessel distributions. Large vessels such as the Icv, SSS, TS and the Lhiv are highlighted due to their consistent presence across different mice, thus serving as landmarks during automatic registration with the images acquired by the standalone OAT system (Fig. 4). The latter can be spatially normalized and coregistered to the CCF, thus enabling ROI-based analysis of the time-lapse (4D) functional OAT data.

To demonstrate robustness of the proposed approach for image registration irrespective of anatomical differences between mice, data from 17 mice of two strains were incorporated into a test OAT dataset with the coregistration performed using methods such as SSD, NCC and MI (see Section 2.7 for details). Initial manual alignment was performed for each OAT image, followed by an automatic registration scheme (Fig. 5a). This consisted of automatic adjustment of an affine transformation considering optimal matching to the OAT dataset after the pre-registration (Fig. 5b). Quantitative evaluation of the registration methods was performed by comparing the RMSE, PSNR and SSIM values calculated between each image and the template following automatic registration versus the respective metrics for manual alignment (see Section 2.7 for details). One OAT image was discarded due to clear misalignment when using SSD or MI. For the remaining images, the calculated RMSE values decreased by 7.54%, 12.66%, and 6.95% for SSD, NCC and MI registration methods, respectively, as compared to the manually aligned images. The corresponding increase in PSNR was 2.65%, 4.47%, and 2.43%, whereas SSIM increased by 17.25%, 30.55%, and 10.89% for the three registration methods, respectively (Fig. 5c). Notably, these findings indicate that the NCC registration method demonstrated the most substantial improvement across all metrics. To investigate the dependency of the optimal registration method on image characteristics, further registration of multiple OAT images to the template was performed using NCC, MI and SSD methods (Fig. 6). For OAT images reconstructed with model-based methods, which demonstrated reduced streak-type artifacts, successful registration was achieved using NCC and MI methods, while SSD registration yielded suboptimal results (Fig. 6a). Conversely, for OAT images reconstructed with backprojection resulting in streak-type artifacts in the background, NCC registration exhibited successful alignment, whereas MI registration displayed suboptimal performance and SSD registration proved unsuccessful (Fig. 6b). Moreover, in the case of OAT images exhibiting a high-intensity artifact, NCC and SSD registrations failed to achieve accurate alignment, whereas MI registration achieved successful results (Fig. 6c).

Brain parcellation of OAT data is straightforward after normalization to the OAT template since the resulting image lies in the Allen CCF (Fig. 7a). In order to further demonstrate the enhanced performance of ROI-based analysis enabled with the template, time-lapse volumetric data was recorded with the standalone OAT imaging system for a mouse subjected to an electrical stimulation paradigm (Fig. 7b). Normalization was performed using a single image frame from the unmixed HbO hemodynamic component. Following manual pre-registration to the OAT template, the image was accurately registered using affine transformations and the NCC registration method. The calculated transformation matrix was then applied to the remaining frames and all the other hemodynamic components. The normalized functional data (with coordinates transformed to the CCF) were analyzed using GLM approach and activation maps were calculated for each hemodynamic component (i.e., HbO, HbR, HbT and sO_2_). Statistically significant activations could then be overlaid onto the Allen brain atlas (Fig. 7c). Significant bilateral activations were observed in the hindlimb area of the primary somatosensory cortex (S1HL) and the primary motor cortex (M1) for all the hemodynamic components. Responses were also detected in major cerebral vessels, such as SSS and TS. Dynamic responses to stimulation were extracted from a 0.3 × 0.3 × 0.3 mm^3^ ROI in the contralateral S1HL region with the signal time-courses averaged across the different stimulation cycles (Fig. 7d). The average HbO, HbT, and sO_2_ signal increase amounted to 7.84 ± 2.21% (SEM), 1.90 ± 1.95%, and 5.66 ± 1.67%, respectively, while the averaged HbR signal intensity decreased by 2.78 ± 1.28%. The time-to-peak (TTP) values of HbO, HbR, HbT and sO2 were calculated as 10 s, 9 s, 7 s and 10 s, respectively, after the stimulation onset. Fractional changes and TTP values were consistent with previous findings [5]. Additionally, functional OAT and MRI data were concurrently recorded with the MROT system under an electrical stimulation paradigm. The functional OAT data were spatially normalized to the Allen CCF and analyzed following the same procedure as for standalone functional OAT data. Statistically significant activations observed in the S1HL and M1 areas exhibited high spatial correlation for the two modalities (Fig. 8).

## 4 Discussion

Accurate navigation and anatomical mapping of the complex mammalian brain is essential for sound interpretation of the information provided by functional neuroimaging modalities. Standardized spaces (brain atlases or templates) are common in neuroimaging studies for accurate analysis and comparison of populations. Brain templates have been reported for several species including humans, rodents, and non-human primates. Anatomical MRI brain atlases are commonly used, and templates for other modalities such as computed tomography and diffusion-weighted imaging have also been reported [1]. Functional OAT has gained significance in neuroimaging studies in the last years owing to its unique capability for simultaneous visualization of multiple hemodynamic components in a label-free and non-invasive fashion. The spectroscopic imaging capacity of OAT is well suited for studying brain disease mechanisms in a preclinical setting e.g. in Alzheimer's or Parkinson's models [39], [40]. However, a standardized OAT mouse brain template featuring distinctive vascular structures visible in the images acquired by different systems has not previously been reported. Our work is the first to provide such a template in the Allen CCF to facilitate accurate region-specific and group-wise data analysis.

OAT-MRI coregistration has been reported using images acquired separately by standalone modalities [29], [30], [31], [32], which was challenging owing to the major differences in the relative positions, orientation and surface deformation of the animals. These effects are minimized when acquiring the bi-modal images concurrently with the hybrid MROT scanner. The developed coregistration methodology can readily be applied to other areas of the body, particularly those most susceptible to deformations such as the abdominal area. Different MRA sequences, namely 2D-TOF-MRA and 3D-FLASH-MRA, were examined to determine the most suitable coregistration protocol. 2D-TOF-MRA can be used without administering contrast agents thus causing less burden on the animals (SFig. **2**). However, the low SNR associated with label-free MRA results in reduced registration performance and the requisite for an additional anatomical scan may present a significant disadvantage particularly if the head motion cannot be fully eliminated. Alternatively, no additional scan is required for OAT-MRI coregistration via 3D-FLASH-MRA, but contrast-enhancement is indispensable for rendering sufficient visibility of the vascular structures.

Intensity-based registration methods, namely SSD, NCC, and MI were exploited for registering OAT images to the template. RMSE and PSNR metrics were combined with SSIM to provide a comprehensive performance assessment and quantify registration quality in terms of noise, error, and perceptual features. Previous work has shown that evaluations solely based on image intensity values (i.e., RMSE and PSNR) may be inconsistent with the human perception, which can be improved with SSIM by taking illumination, contrast, and structure into consideration [37]. Quantitative evaluation of the three registration methods revealed that NCC scored the best in terms of registration accuracy, as evinced by the highest percentile change observed across all performance evaluation metrics. Since OAT images primarily visualize the hemoglobin distribution within the brain, major vessels correspond to higher intensity values. This may arguably explain the greater accuracy achieved with the NCC registration, as the contribution of a given voxel to the final result is heavily dependent on its intensity value in correlation-based registration methods. However, the NCC registration may turn ineffective when high-intensity artifacts are present in the image [41]. The SSD registration method assumes instead uniform intensities across the images performing optimally when the images differ only by Gaussian noise. This is hardly the case for OAT images due to differences in output laser pulse energy, animal positioning, and tomographic acquisition geometries across systems. In contrast, entropy-based methods like MI registration measure general dependences between images rather than assuming a linear dependence. MI registration is consequently less sensitive to minute high-intensity artifacts but it fails when slowly varying intensity is manifested in the background [41]. Therefore, streak-type artifacts, commonly observed in OAT images rendered with spatially undersampled tomographic data [42], may hinder the performance of MI registration. The optimal registration method thus heavily depends on the image characteristics, such as its noise level and type of artifacts present (Fig. 6). Moreover, it is crucial that the spatial resolution of the standalone OAT system approximately matches that of the MROT system, in a way that the essential structures of the template (e.g. superior sagittal sinus and vein of Galen) are easily discernible. In this manner, proper registration of images acquired with different OAT systems can be achieved.

It is important to note that the suggested OAT template method has several limitations. First, the hybrid MROT scanner was designed to fit into the MRI bore and has a limited effective field-of-view that may hinder accurate representation of vessels in the peripheral brain regions. Furthermore, the template was constructed using a single mouse strain with a small number of mice (N = 11), which may limit its applicability. However, we did not observe any obvious difference in registration accuracy between the two mouse strains included in the test OAT dataset nor when increasing the mouse number from 6 to 11 for the template development. Lastly, due to the limited penetration depth of OAT, primarily caused by strong light attenuation in vascularized tissues, the template mainly covers the cortical and hippocampal brain regions.

## 5 Conclusion

In conclusion, we report on the first OAT mouse brain template that takes advantage of the recently developed hybrid MROT imager with simultaneous OAT and MRI data acquisition. By registering OAT images acquired from standalone systems to the brain template (already coregistered with the Allen mouse brain atlas), accurate brain parcellation and anatomical referencing can be achieved as evinced by our functional stimulus-evoked neuroimaging study. The proposed method thus helps transform structural or functional OAT data from an arbitrary subject space defined by a custom-designed system to a standard common space. This in turn enables better consolidation of the research findings hence facilitating wider acceptance of OAT as a powerful neuroimaging tool to study brain functions and diseases.

## CRediT authorship contribution statement

ZC conceived the concept. IG, ZC and HY performed the animal experiments. IG performed data analysis. ZC, XLD provided guidance on experimental procedures and data analysis. DR supervised the work. All authors contributed to writing and revising the manuscript.

## Data and code availability statement

The constructed OAT template with the coregistered MRI atlas, the test OAT dataset and the activation maps of the functional OAT data are available online (https://doi.org/10.3929/ethz-b-000607219). The code that supports the findings of this study are available from the corresponding author upon reasonable request.

## Declaration of Competing Interest

The authors declare that they have no known competing financial interests or personal relationships that could have appeared to influence the work reported in this paper.

## Data Availability

Data will be made available on request.

## References

[1] Mandal P.K., Mahajan R., Dinov I.D. (2012). Structural brain atlases: design, rationale, and applications in normal and pathological cohorts. J. Alzheimers Dis..

[2] Schwarz A.J. (2006). A stereotaxic MRI template set for the rat brain with tissue class distribution maps and co-registered anatomical atlas: application to pharmacological MRI. Neuroimage.

[3] Chen Z.Y. (2022). Hybrid magnetic resonance and optoacoustic tomography (MROT) for preclinical neuroimaging. Light-Sci. Appl..

[4] Chen Z.Y. (2022). Multimodal noninvasive functional neurophotonic imaging of murine brain-wide sensory responses. Adv. Sci..

[5] Chen, Z.Y., et al., Simultaneous Functional Magnetic Resonance and Optoacoustic Imaging of Brain-Wide Sensory Responses in Mice. Advanced Science, 2022. 10.1002/advs.202205191 PMC9875624 36437110

[6] Vu T., Razansky D., Yao J. (2019). Listening to tissues with new light: recent technological advances in photoacoustic imaging. J. Opt..

[7] Choi W. (2018). Clinical photoacoustic imaging platforms. Biomed. Eng. Lett..

[8] Lin L., Wang L.V. (2022). The emerging role of photoacoustic imaging in clinical oncology. Nat. Rev. Clin. Oncol..

[9] Laufer J. (2012). In vivo preclinical photoacoustic imaging of tumor vasculature development and therapy. J. Biomed. Opt..

[10] Dean-Ben X., Fehm T.F., Razansky D. (2014). Universal hand-held three-dimensional optoacoustic imaging probe for deep tissue human angiography and functional preclinical studies in real time. J. Vis. Exp..

[11] Li M.L. (2008). Simultaneous molecular and hypoxia imaging of brain tumors in vivo using spectroscopic photoacoustic tomography. Proc. Ieee.

[12] Dean-Ben X.L., Razansky D. (2021). Optoacoustic imaging of the skin. Exp. Dermatol..

[13] Wang B. (2010). Detection of lipid in atherosclerotic vessels using ultrasound-guided spectroscopic intravascular photoacoustic imaging. Opt. Express.

[14] Kim G. (2007). Indocyanine-green-embedded PEBBLEs as a contrast agent for photoacoustic imaging. J. Biomed. Opt..

[15] Razansky D. (2009). Multispectral opto-acoustic tomography of deep-seated fluorescent proteins in vivo. Nat. Photonics.

[16] Tomaszewski M.R. (2017). Oxygen enhanced optoacoustic tomography (OE-OT) reveals vascular dynamics in murine models of prostate cancer. Theranostics.

[17] Ku G. (2005). Imaging of tumor angiogenesis in rat brains in vivo by photoacoustic tomography. Appl. Opt..

[18] Manohar S., Dantuma M. (2019). Current and future trends in photoacoustic breast imaging. Photoacoustics.

[19] Guo Q., Wang D., Yang G.L. (2019). Photoacoustic imaging guided photothermal and chemodynamic combined therapy for cancer using "all in one" W18O49 nanorod agent. J. Biomed. Nanotechnol..

[20] Bohndiek S.E. (2015). Photoacoustic tomography detects early vessel regression and normalization during ovarian tumor response to the antiangiogenic therapy trebananib. J. Nucl. Med..

[21] Mallidi S. (2015). Prediction of Tumor Recurrence and Therapy Monitoring Using Ultrasound-Guided Photoacoustic Imaging. Theranostics.

[22] Mc Larney B. (2020). Monitoring of stimulus evoked murine somatosensory cortex hemodynamic activity with volumetric multi-spectral optoacoustic tomography. Front. Neurosci..

[23] Gottschalk S. (2019). Rapid volumetric optoacoustic imaging of neural dynamics across the mouse brain. Nat. Biomed. Eng..

[24] Rich L.J., Seshadri M. (2016). Photoacoustic monitoring of tumor and normal tissue response to radiation. Sci. Rep..

[25] Chen Z.Y. (2019). Concurrent fluorescence and volumetric optoacoustic tomography of nanoagent perfusion and bio-distribution in solid tumors. Biomed. Opt. Express.

[26] Özsoy Ç. (2022). Volumetric optoacoustic neurobehavioral tracking of epileptic seizures in freely-swimming zebrafish larvae. Front. Mol. Neurosci..

[27] Lafci B. (2020). Noninvasive multiparametric characterization of mammary tumors with transmission-reflection optoacoustic ultrasound. Neoplasia.

[28] Ni R. (2021). In-vitro and in-vivo characterization of CRANAD-2 for multi-spectral optoacoustic tomography and fluorescence imaging of amyloid-beta deposits in Alzheimer mice. Photoacoustics.

[29] Attia A.B. (2016). Multispectral optoacoustic and MRI coregistration for molecular imaging of orthotopic model of human glioblastoma. J. Biophoton..

[30] Gehrung M. (2020). Co -registration of optoacoustic tomography and magnetic resonance imaging data from murine tumour models. Photoacoustics.

[31] Ren W. (2019). Automated registration of magnetic resonance imaging and optoacoustic tomography data for experimental studies. Neurophotonics.

[32] Hu, Y., et al., Automatic image registration of optoacoustic tomography and magnetic resonance imaging based on deep learning. First Conference on Biomedical Photonics and Cross-Fusion (BPC 2022). Vol. 12461. 2022: SPIE.

[33] Xu M.H., Wang L.H.V. (2005). Universal back-projection algorithm for photoacoustic computed tomography. Phys. Rev. E.

[34] Wang, Q.X., et al., The Allen Mouse Brain Common Coordinate Framework: A 3D Reference Atlas. Cell, 2020. 181(4): p. 936-+. 10.1016/j.cell.2020.04.007 PMC8152789 32386544

[35] Oliveira F.P.M., Tavares J.M.R.S. (2014). Medical image registration: a review. Comput. Methods Biomech. Biomed. Eng..

[36] Hill D.L. (2001). Medical image registration. Phys. Med Biol..

[37] Sara U., Akter M., Uddin M. (2019). Image quality assessment through FSIM, SSIM, MSE and PSNR—a comparative study. J. Comput. Commun..

[38] Lambers H. (2020). A cortical rat hemodynamic response function for improved detection of BOLD activation under common experimental conditions. Neuroimage.

[39] Ni R. (2022). Multiscale optical and optoacoustic imaging of amyloid-β deposits in mice. Nat. Biomed. Eng..

[40] Ovsepian S.V. (2017). Pushing the boundaries of neuroimaging with optoacoustics. Neuron.

[41] Penney G.P. (1998). A comparison of similarity measures for use in 2-D-3-D medical image registration. Ieee Trans. Med. Imaging.

[42] Dean-Ben X.L. (2012). Accurate model-based reconstruction algorithm for three-dimensional optoacoustic tomography. Ieee Trans. Med. Imaging.

