# Supplementary material for: Volumetric registration framework for multimodal functional magnetic resonance and optoacoustic tomography of the rodent brain
Source: Photoacoustics. 2023 Jun 9;31:100522. doi: 10.1016/j.pacs.2023.100522 (PMC10285284; doi:10.1016/j.pacs.2023.100522)
Supplement: Supplementary file 1 — Supplementary material [file mmc1.docx]

**Volumetric Registration Framework for Multimodal Functional Magnetic Resonance and Optoacoustic Tomography of the Rodent Brain**

Irmak Gezginer^1,2^, Zhenyue Chen^1,2^, Hikari A. I. Yoshihara^1,2^, Xosé Luís Deán-Ben^1,2^, and Daniel Razansky^1,2,*^

^1^ Institute for Biomedical Engineering and Institute of Pharmacology and Toxicology, Faculty of Medicine, University of Zurich, Switzerland

^2^ Institute for Biomedical Engineering, Department of Information Technology and Electrical Engineering, ETH Zurich, Switzerland

*Correspondence

Daniel Razansky, Institute for Biomedical Engineering, Wolfgang-Pauli-Strasse 27, CH-8093 Zurich, Switzerland

Email: daniel.razansky@uzh.ch

**Supplementary Material**

**
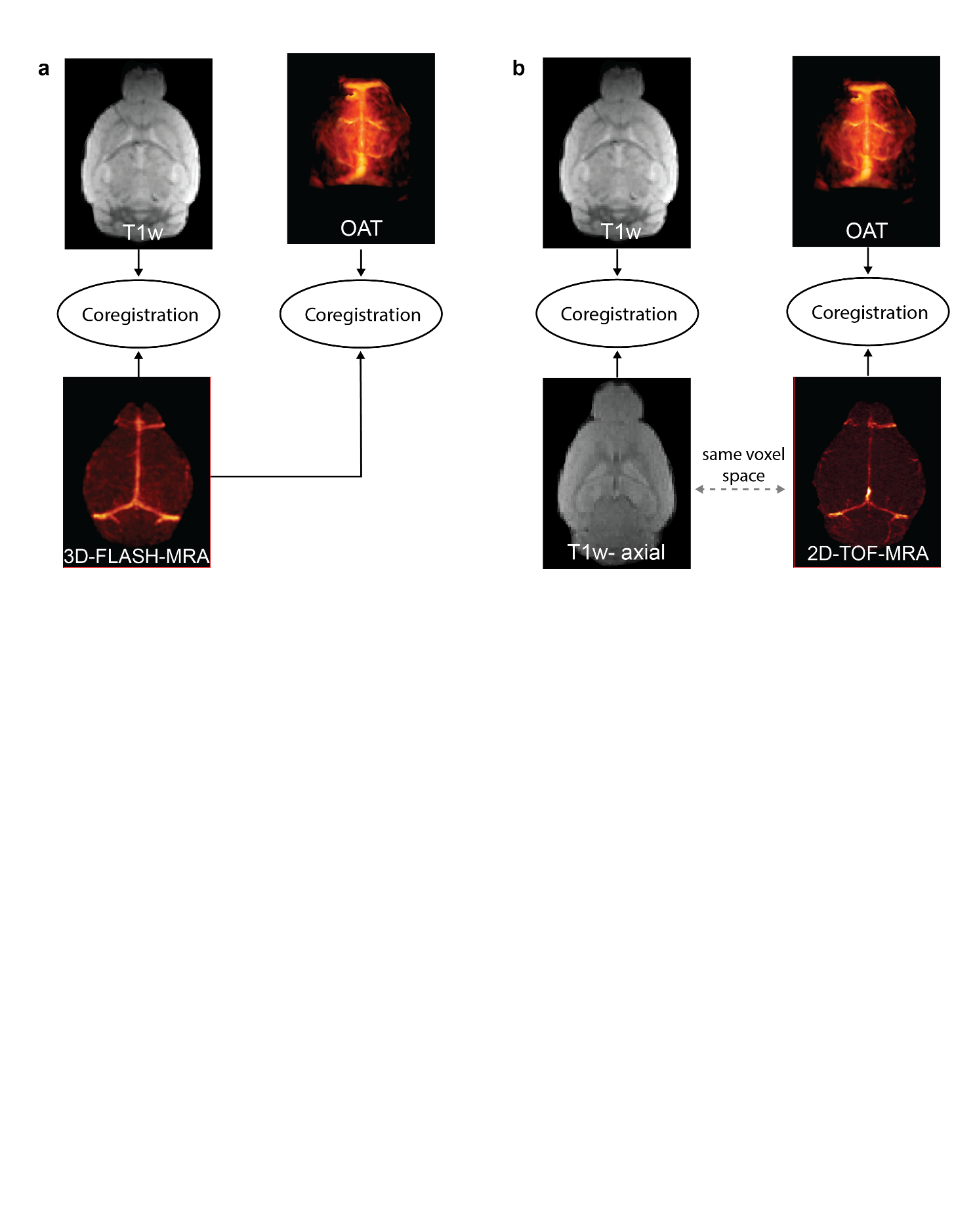
**

**Supplementary Figure 1:** Coregistration of OAT and MRI data with two different methods. **a** OAT-MRI coregistration using 3D-FLASH-MRA. Coregistration of isotropic anatomical MRI image (T1w) to 3D-FLASH-MRA is followed by coregistration of OAT image to 3D-FLASH-MRA. Both processes involve an initial manual alignment, followed by automatic alignment using mutual information. **b** OAT-MRI coregistration using 2D-TOF-MRA. Due to the lack of soft tissue contrast of 2D-TOF-MRA, an anatomical scan (T1w-axial) lying in the same coordinate space as the MRA is additionally employed. Coregistration of the anatomical scans and OAT image to 2D-TOF-MRA are subsequently realized. Both processes consist of automatic alignment of images using mutual information following an initial manual alignment.

**
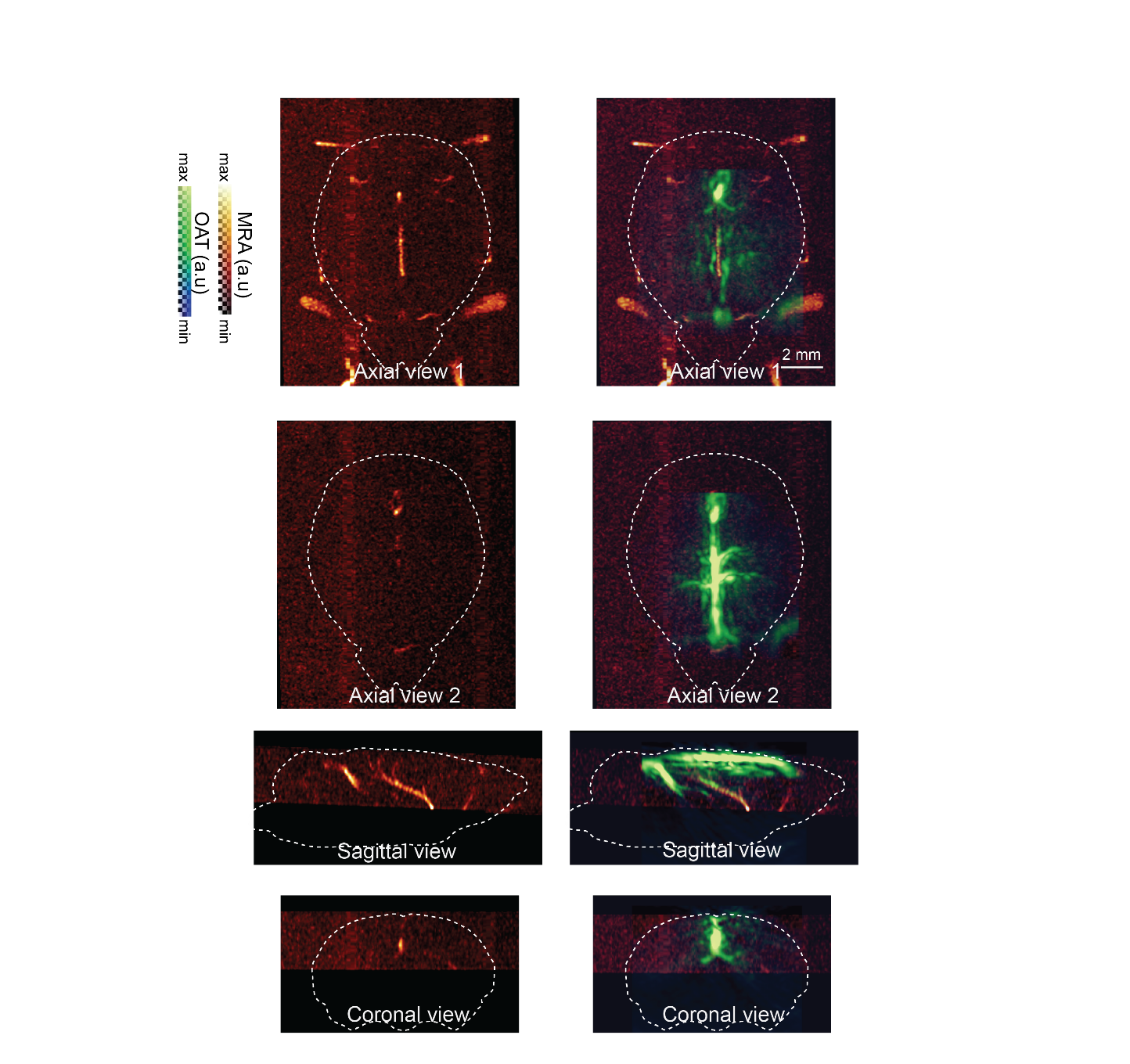
**

**Supplementary Figure 2:** Coregistration of OAT and 2D-TOF-MRA images without contrast enhancement acquired with the MROT scanner. Axial, sagittal and coronal views of 2D-TOF-MRA image superposed to the coregistered OAT image. Images are shown as MIPs of a 0.8 mm thick volumes.
